# Menstrual migraine, migraine and contraceptions, migraine and pregnancy and migraine triggers

**DOI:** 10.1186/1129-2377-14-S1-O5

**Published:** 2013-02-21

**Authors:** Anne MacGregor

**Affiliations:** 1 Barts Sexual Health Centre, St Bartholomew's Hospital, London, UK

## 

More than 50% of women with migraine report an association with menstruation. Attacks are most likely to occur during the two days before menstruation and the first three days of bleeding and are typically without aura, even in women who have attacks with aura at other times of the cycle. The majority of menstrual attacks can be controlled with symptomatic treatment alone. If this is inadequate, pre-emptive treatment with perimenstrual estradiol, triptans or non-steroidal anti-inflammatory drugs (NSAIDs) may be effective.

Suppression of the menstrual cycle with anovulatory contraceptive agents is an additional option for management, particularly for women who also require contraception. Using combined hormonal contraception continuously, without a break, can prevent estrogen “withdrawal” migraine during the hormone-free interval.

Limited research suggests that high levels of estrogen, such as occur with use of combined hormonal contraceptives (CHCs), can trigger migraine with aura. Since aura and CHCs are independent risk factors for ischaemic stroke, the appropriateness of CHCs solely for contraception in women who have migraine with aura needs careful consideration.

During pregnancy, migraine follows a benign course with improvement in migraine without aura likely by the second trimester. However, the high oestrogen state is associated with increased migraine aura in susceptible women. In these women, hypertensive disorders of pregnancy and stroke are more likely to occur although the absolute risk remains small. Women benefit from early advice on drugs that can be continued during pregnancy in order to optimize control and avoid unnecessary concern

